# A Bibliometric Analysis on Research Trends in Paediatric Epilepsy Treatment From 2005 to 2025

**DOI:** 10.7759/cureus.87688

**Published:** 2025-07-10

**Authors:** Oliver Squires

**Affiliations:** 1 Paediatrics, Perth Children's Hospital, Perth, AUS

**Keywords:** anti-epileptic, childhood, intervention, management, seizure, therapy

## Abstract

The objective of this bibliometric analysis was to evaluate trends in paediatric epilepsy treatment research from 2005 to 2025. Through assessing which journals, countries, authors, and institutions have been the most productive and mapping trends in keywords used over time, the study aims to reveal emerging areas of interest and inspire ideas for future research. A total of 1418 relevant documents were retrieved from the Web of Science database. Documents outside of the target time period and non-English documents were excluded. From the 1152 remaining documents, 17 duplicates were removed using OpenRefine (OpenRefine). The final 1135 documents were then analysed using VosViewer (VOSviewer - Visualizing scientific landscapes) and Bibliometrix (https://www.bibliometrix.org/).

The analysis revealed a stable number of annual global publications until a notable increase since 2018. The USA leads the way with the greatest research output (298 articles) and the highest number of total citations (6565). Harvard University emerged as the most productive institution (111 articles), whilst Epilepsia was the most productive journal (253 articles). Keyword co-occurrence analysis revealed a shift in research from earlier documents focusing more on traditional drug therapies to more recent articles focusing on cannabidiol based therapies, laser ablation, and vagal nerve stimulation.

This bibliometric analysis provides new insights into the evolution of paediatric epilepsy treatment research. It helps to demonstrate the leading contributors in the field over the last 20 years, as well as highlighting emerging new trends. The results of the analysis underline the shift in the research away from more traditional treatments to investigating alternative therapies, including cannabidiol based therapies, dietary interventions, laser ablation, and vagal nerve stimulation. Through identifying these trends in the research and mapping author collaboration networks, it is hoped that this study can provide a foundational reference for researchers when planning future studies.

## Introduction and background

Epilepsy is defined as a brain disorder in which the affected individual has an ongoing increased risk of experiencing epileptic seizures [1]. It affects an estimated 0.5% to 1% of children, making it the most common chronic neurological condition seen in childhood [2]. It is associated with a range of morbidities, including accidents that occur during seizures, aspiration pneumonias, direct brain injury from prolonged seizures, and Sudden Unexplained Death in Epilepsy [3]. Individuals with epilepsy live an estimated two to 11 years less than the rest of the population [4,5].

The management of epilepsy in children presents unique challenges. Diagnostic difficulty due to broad clinical manifestations of the condition, developmental considerations, and associated behavioural and cognitive co-morbidities are all contributing factors [6]. Consequently, it becomes apparent that research into efficacious treatments for epilepsy is essential for improving the quality and length of life of children with the condition. Over the past two decades, notable progress has been made in the understanding and management of epilepsy. Advances have been made in surgical interventions, antiepileptic drugs as well as non-pharmacological interventions. As therapeutic options continue to evolve, it becomes important to examine the field to uncover research gaps and identify emerging areas of interest.

Bibliometric analysis is a powerful method that involves retrospective analysis of large amounts of scientific data to elucidate research trends in that area as well as calculating objective markers of performance such as the number of publications and citations. It can be useful for highlighting relationships between collaborating authors, which countries and institutions have formed close links over time, and which journals and authors have been the most productive in a particular field [7]. The aim of this review was to objectively quantify the most productive journals, institutions, countries, and authors in the field as well as conduct keyword co-occurrence with the hope that emerging trends in the research could be identified and directions for new research could be inspired. This bibliometric analysis aims to support the advancement of therapeutic options for treating epilepsy and, ultimately, improve outcomes for children with the condition.

## Review

Methods

Web of Science (WOS) is one of the largest and most comprehensive databases of bibliometric data available and is frequently used in bibliometric analyses [8]. As a result, WOS was the only source of data used in this review. The identification and screening of appropriate records for this analysis were carried out in accordance with the Preferred Reporting Items for Systematic Reviews and Meta-Analyses (PRISMA) guidelines [9]. To identify a suitable sample of documents relevant to the analysis the following search strategy was applied: ("paediatric" OR “pediatric" OR "childhood" OR "child" OR "children") AND ("epilepsy" OR "status epilepticus") AND ("management" OR "treatment" OR “therapy”). This search strategy was applied to the title of documents only and yielded 1418 results when carried out on the 28/01/2025. Two hundred seventeen documents were excluded for being outside of the target time period of 01/01/2005-28/01/2025. Forty-nine documents that were not in English were excluded.

OpenRefine (OpenRefine) is a powerful open-source tool that allows large datasets to be cleaned, organised, and duplicate studies to be removed [10]. In this analysis, this tool was primarily used to organise the data, cluster appropriate terms, and remove 17 duplicate documents. This resulted in 1135 documents being included in the final bibliometric analysis that was carried out using VosViewer (VOSviewer - Visualizing scientific landscapes) and Bibliometrix (https://www.bibliometrix.org/). VosViewer is a computer program commonly used in Bibliometric analyses that allows large amounts of data to be analysed using an algorithm to form clusters of related terms and maps formed of links between different countries, institutions, journals, and authors based on relationships between citations [11]. Bibliometrix is another tool that can be used to carry out comprehensive science mapping analysis. It allows large volumes of data to be analysed and visualised [12].

**Figure 1:**
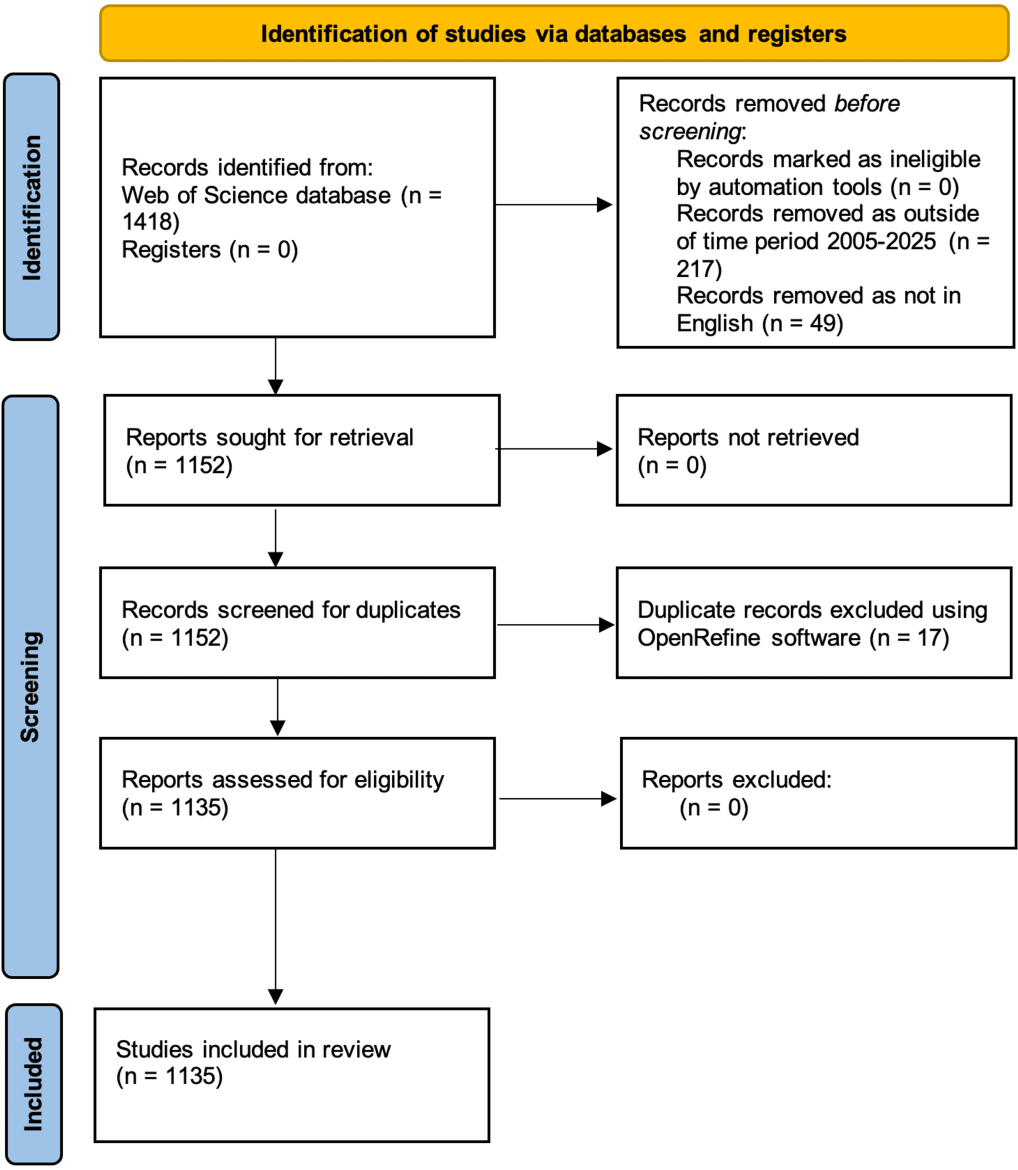
PRISMA flow chart to demonstrate the selection process for articles included in this study The flow chart is created by the author based on the 2020 Preferred Reporting Items for Systematic Reviews and Meta-Analyses (PRISMA) guidelines.

Results

Global Publication Changes Over Time

**Figure 2:**
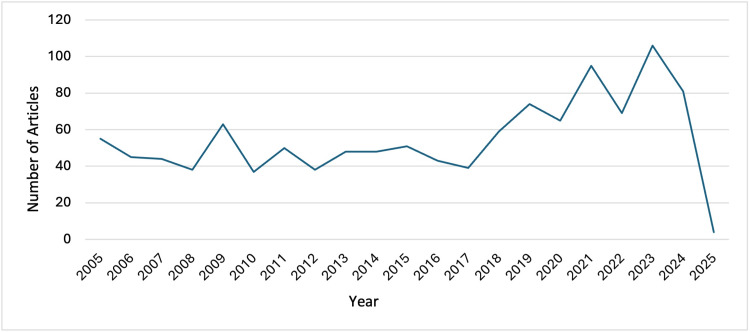
A graph to show the number of articles published per year The graph is created by the author.

Throughout the last 20 years, the number of yearly publications has remained at a relatively constant level until a notable increase from 2018 onwards (aside from 2025 so far, for which only the first 28 days have been included in this analysis). The most productive year was 2023, during which 106 documents were published, as shown in Figure 2. 

Geographical Variability in Publications

**Figure 3:**
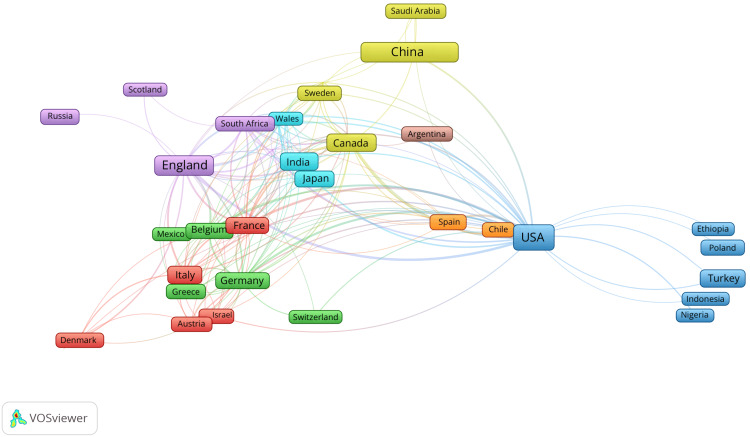
A co-authorship analysis map showing links between the countries of different authors The image was created bythe author using the VOSviewer software (available at https://www.vosviewer.com).

**Figure 4:**
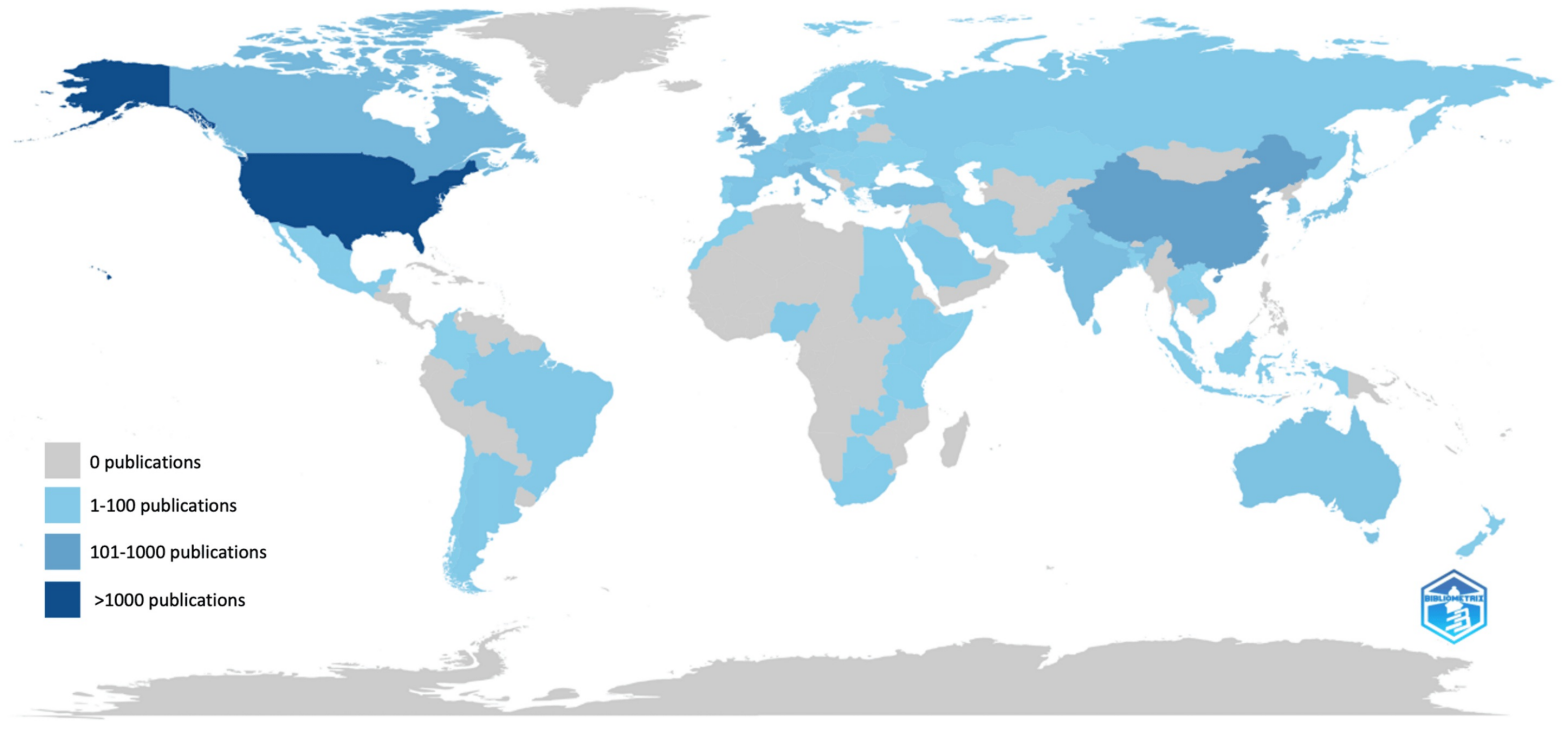
A map reflecting the number of articles published by different countries across the globe The image was generated by the author using the Bibliometrix package (v3.0.4; https://www.bibliometrix.org/) [12]. A legend was then manually added to the exported image by the author.

As displayed in Table 1, and Figures 3, 4, the USA was the most productive country during this period, producing 298 articles, followed by the Republic of China with 109 articles and then the United Kingdom (UK) with 62 articles. The USA also led the way with the most total citations (6565), followed by the UK (2653) and then China (899). The USA also collaborated the greatest number of times, with 32 documents involving collaboration between authors of different countries. Germany had the highest percentage of ‘multiple country publications’ (47.1%), and South Korea had the least (0%). In the co-authorship map below, the size of the labels represents the number of publications of each country, and the lines represent links between countries collaborating.

**Table 1:** Geographical variability in country of publication SCP, single country publication; MCP, multiple country publication. N = 1135. Note: only the top 10 countries according to the number of published articles are displayed in this table.

Country	Articles	Articles %	Total Citations	SCP	MCP	MCP %
USA	298	26.3	6565	266	32	10.7
China	109	9.6	899	102	7	6.4
United Kingdom	62	5.5	2653	48	14	22.6
Italy	43	3.8	770	39	4	9.3
Korea	43	3.8	479	43	0	0
Turkey	38	3.3	495	37	1	2.6
Canada	33	2.9	700	25	8	24.2
India	31	2.7	488	30	1	3.2
Japan	22	1.9	252	21	1	4.5
Germany	17	1.5	333	9	8	47.1

Institution Affiliations

The institution associated with the most publications was Harvard University, with 111 articles, followed by the University College London with 99 articles, and then Harvard Medical School Affiliates with 79 articles, as shown in Table 2.

**Table 2:** Top 10 institutions by number of articles published

Affiliation	Country	Articles
Harvard University	USA	111
University College London	UK	99
Harvard Medical School Affiliates	USA	79
Boston Children's Hospital	USA	75
University of Toronto	Canada	69
Cincinnati Children's Hospital Medical Center	USA	60
Harvard Medical School	USA	55
University System of Ohio	USA	52
University of Pennsylvania	USA	51
Hospital for Sick Children (sickkids)	Canada	41

Journals

Table 3 shows that the most productive journal was Epilepsia with 250 documents, followed by Epilepsy & Behavior with 63 documents, and then Seizure-European Journal of Epilepsy with 55 documents. Epilepsia also led the way with 4448 local citations, followed by Neurology with 1624, then Epilepsy & Behavior with 1564. ‘Total citations’ refers to the total number of times an article has been cited by all sources in comparison to ‘local citations’ that represents the number of times that an article has been cited only by other articles included within the analysis. Local citations can give a more relevant indication of a document’s influence within the area of research being studied.

**Table 3:** Top 10 Journals with highest number of publications and number of local citations

Journal	Articles	Local Citations
Epilepsia	253	4448
Epilepsy & Behavior	63	1564
Seizure - European Journal of Epilepsy	56	1130
Neurology	39	1624
Journal of Child Neurology	33	681
Pediatric Neurology	30	605
Epilepsy Research	23	852
Journal of Neurosurgery - Pediatrics	22	188
Annals of Neurology	21	372
Epileptic Disorders	19	233

Authors

**Figure 5:**
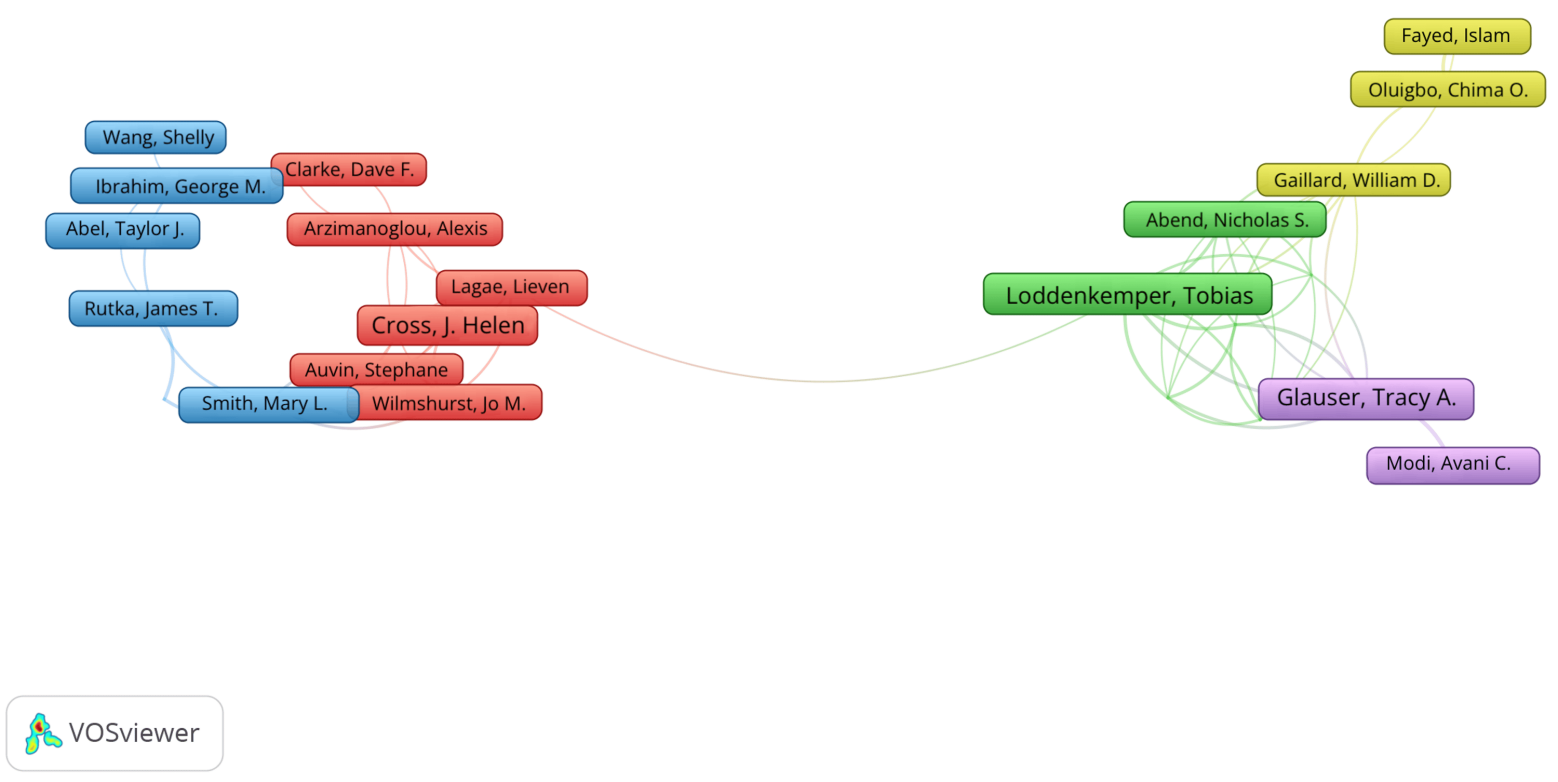
A co-authorship map showing relationships between authors The image was created by the author using the VOSviewer software (available at https://www.vosviewer.com).

Table 4 and Figure 5 demonstrate that the most productive author during this period was Tobias Loddenkemper with 23 publications, followed by Tracey A. Glauser with 20, and then J. Helen Cross with 17. Tracey A. Glauser had the greatest number of total citations (1580) followed by J. Helen Cross (1318) then Avani C. Modi (410). Tracey A. Glauser had the highest h-index (14). Clusters of authors co-authoring publications were also analysed, and only those with at least five publications were included when generating maps using VosViewer. In the map below, lines between names indicate co-authorship of papers, with shorter distances between names indicating greater relatedness. Five clusters of closely collaborating authors were revealed, indicated by labels of different colours. The cluster involving the most authors included J. Helen Cross, Dave F. Clarke, Alexis Arzimanoglou, Lieven Lagae, Stephane Auvin, and Jo M. Wilmshurt.

**Table 4:** Top 10 authors ordered by number of total publications

Author	Institution	Country	Total Publications	Total citations	h_index	g_index	m_index
Loddenkemper, Tobias	Harvard University	USA	23	408	10	20	0.625
Glauser, Tracy A.	Cincinnati Children’s Hospital	USA	20	1580	14	20	0.7
Cross, J. Helen	University College London	England	17	1318	11	17	0.611
Modi, Avani C.	Cincinnati Children’s Hospital	USA	12	410	7	12	0.35
Abend, Nicholas S.	University of Pennsylvania	USA	11	353	9	11	0.563
Kim, Heung-Dong	Yonsei University	South Korea	11	200	8	11	0.4
Tasker, Robert C.	Harvard Medical School	USA	9	275	6	9	0.462
Gaillard, William D.	George Washington University	USA	8	223	6	8	0.4
Rutka, James T.	The Hospital for Sick Children	Canada	8	252	6	8	0.353
Abel, Taylor J.	University of Pittsburgh	USA	7	143	5	7	0.714

Top Cited Articles

Table 5 shows that the article with the greatest number of total citations (767) was ‘the ketogenic diet for the treatment of childhood epilepsy: a randomised controlled trial’ from 2008 with Elizabeth G. Neal as the first author. The article with the greatest number of ‘total citations per year’ (76.40) was ‘Evidence-Based Guideline: Treatment of Convulsive Status Epilepticus in Children and Adults: Report of the Guideline Committee of the American Epilepsy Society’ from 2016, with Tracey A. Glauser as the first author.

**Table 5:** Top 10 most cited articles

First Author	Title	Year	Total Citations	Total Citations per Year
Neal, Elizabeth G.	The ketogenic diet for the treatment of childhood epilepsy: a randomised controlled trial	2008	767	42.61
Glauser, Tracy A.	Evidence-Based Guideline: Treatment of Convulsive Status Epilepticus in Children and Adults: Report of the Guideline Committee of the American Epilepsy Society	2016	764	76.40
Neal, Elizabeth G.	A randomized trial of classical and medium-chain triglyceride ketogenic diets in the treatment of childhood epilepsy	2009	285	16.76
Porter, Brenda E.	Report of a parent survey of cannabidiol-enriched cannabis use in pediatric treatment-resistant epilepsy	2013	237	18.23
Kossoff, Eric H.	A Modified Atkins Diet Is Effective for the Treatment of Intractable Pediatric Epilepsy	2006	235	11.75
Kramer, Uri.	Febrile infection–related epilepsy syndrome (FIRES): Pathogenesis, treatment, and outcome	2011	223	14.87
Wheless, James W.	Treatment of pediatric epilepsy: European expert opinion, 2007	2007	217	11.42
Modi, Avani C.	Patterns of Nonadherence to Antiepileptic Drug Therapy in Children With Newly Diagnosed Epilepsy	2011	181	12.07
Chin, Richard F.M.	Treatment of community-onset, childhood convulsive status epilepticus: a prospective, population-based study	2008	177	9.83
Wheless, James W.	Treatment of Pediatric Epilepsy: Expert Opinion, 2005	2005	164	7.81

Keyword Co-occurrence

**Figure 6:**
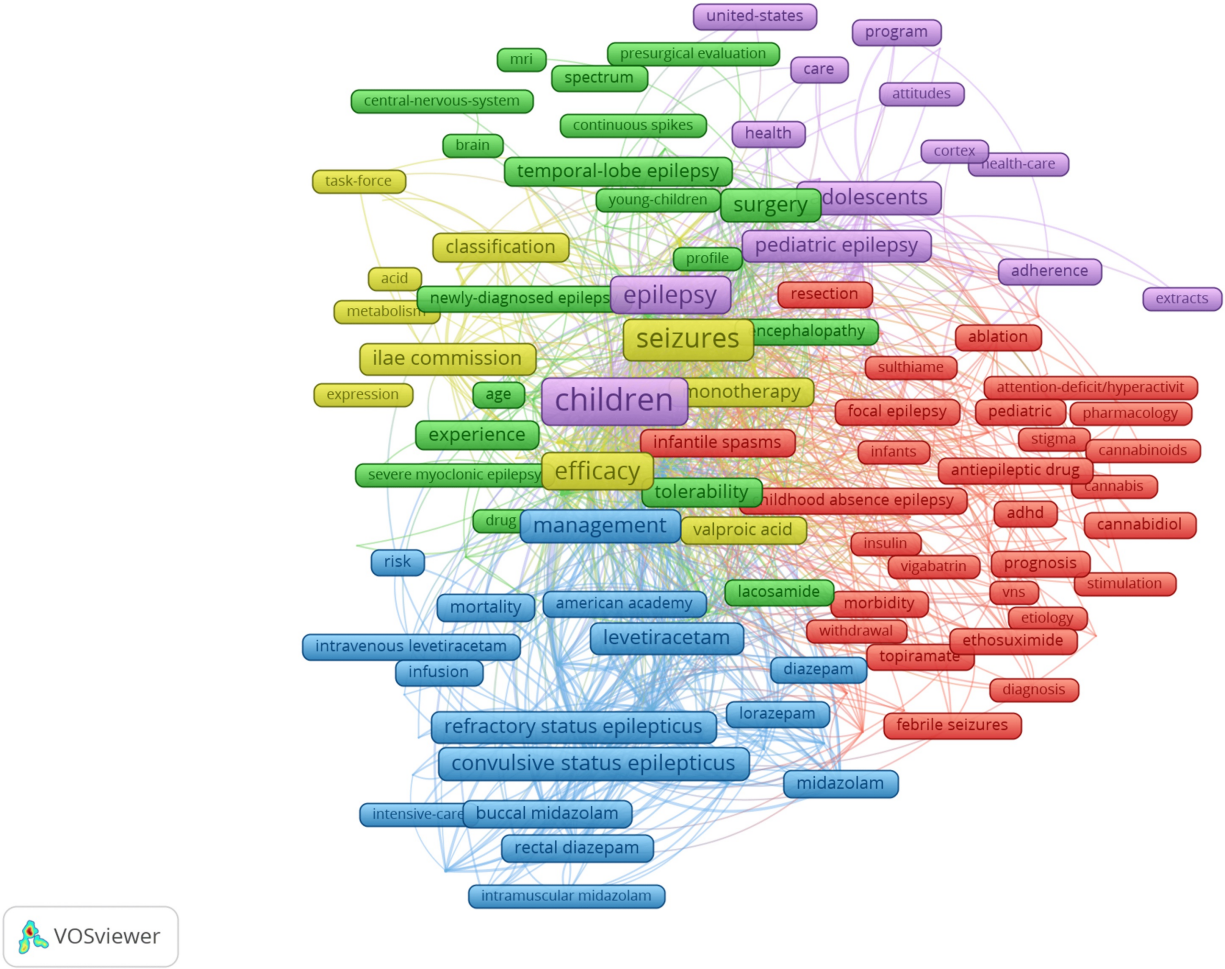
A keyword co-occurrence map showing the most common words used and their relationship with other commonly occurring words The image was created by the author using the VOSviewer software (available at https://www.vosviewer.com).

**Figure 7:**
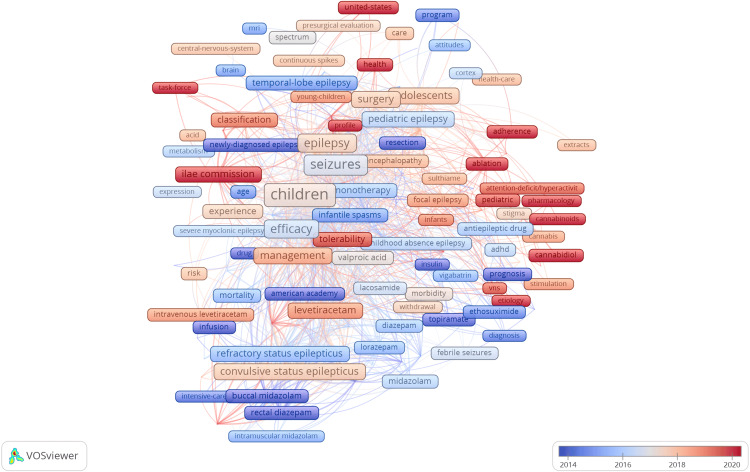
A keyword co-occurrence map showing changes in trends of commonly used keywords over time The image was created by the author using the VOSviewer software (available at https://www.vosviewer.com).

Keyword co-occurrence analysis is displayed in Table 6 and Figures 6, 7. In a keyword co-occurrence map, lines between words indicate that they commonly occur in documents together. The size of the label represents how commonly that word occurs, with a larger label indicating a more frequently occurring word. Clusters of words commonly appearing together are represented by the different colours in the map below [10]. The most common keywords were ‘children’ (317 occurrences), followed by ‘epilepsy’ (269 occurrences) and then ‘seizures’ (185 occurrences). The keywords most commonly featured in these documents have evolved throughout time. Some of the most common keywords in older publications were ‘buccal midazolam’, ‘rectal diazepam’, ‘topiramate’, ‘insulin’, ‘ethosuximide’ and ‘resection’. The most common keywords in more recent publications included ‘VNS’, ‘stimulation’, ‘cannabinoids’, cannabidiol’, ‘cannabis’, ‘attention-deficit/hyperactivity disorder’, ‘ablation’, ‘surgery’, ‘sulthiame’ and ‘intravenous levetiracetam’.

**Table 6:** Top 10 most common keywords

Keyword	Occurrences
Children	317
Epilepsy	269
Seizures	185
Efficacy	125
Antiepileptic drugs	87
Ketogenic diet	84
Refractory epilepsy	84
Status epilepticus	82
Convulsive status epilepticus	69
Management	68

Discussion

This bibliometric analysis is the first to cover research into treatments for paediatric epilepsy. The findings of this analysis help to highlight trends in the field over the last 20 years, show clusters of collaborating authors, and allow for the identification of new avenues of research.

Authors from the USA have led the way throughout this period, contributing the largest number of publications by a significant margin as well as have the largest number of citations. Seven out of the ten most productive authors work out of institutions in the USA, the exceptions being Helen Cross (University College London, England), Kim Heung-Dong (Yonsei University, South Korea) and James T. Rutka (The Hospital for Sick Children, Canada), all with notable contributions to the field. Tobias Loddenkemper was the most productive individual author throughout this period with 23 publications, whereas the author with the highest impact was Tracey Glauser with an h-index of 14, 1580 total citations, and regular publications over the years. This dominance of the USA reflects its historically strong research infrastructure and that it invests more in research and development than any other country [13].

Amongst the top 10 most productive countries, the country showing the highest degree of international collaboration was Germany with an MCP% (multiple country publications percentage) of 47.1% in contrast with South Korea, which had an MCP% of 0% indicating none of the articles included in this review involved inter-country collaboration. This broadly mirrors patterns documented previously with European countries, such as Germany, which collaborates more frequently. This has often been put down to their geographical proximity to other nations involved with research. South Korea has shown gradually decreasing levels of international cooperation in research that has been attributed previously to rising levels of development throughout South Korea and its research institutes and a subsequent decrease in reliance on international research partners [14].

Keyword co-occurrence maps help to highlight shifting trends in the research over time. Earlier research articles more commonly focused on benzodiazepines with ‘buccal midazolam’, ‘rectal diazepam’, ‘lorazepam’ and ‘intramuscular midazolam’ amongst the most common words. In contrast to this, more recent studies frequently mention ‘cannabidiol’ (CBD), ‘cannabinoids’, ‘cannabis’, ‘ablation’ and ‘VNS’ (vagus nerve stimulation). Antiepileptic drugs are widely recommended by organisations such as the National Institute for Health and Care Excellence (NICE) as the first-line treatment for epilepsy in children [15]. Lamotrigine, levetiracetam, and topiramate have been demonstrated as being the most efficacious antiepileptic drugs in terms of patients becoming seizure-free. Even so, more high-quality randomised controlled trials, especially involving children younger than two years old, have been considered necessary [16]. 

The increase in research related to CBD has been seen throughout scientific literature, with the number of published articles increasing rapidly, especially from 2019 onwards [17]. This could be related to increasing societal acceptance and interest in CBD as a therapeutic agent and the approval of a CBD-related drug in 2018 by the FDA for the treatment of epilepsy in children with Dravet syndrome [18]. Multiple randomised controlled trials have been carried out, confirming the efficacy of CBD in reducing seizure frequency in children with refractory epilepsy. These studies have also revealed that, although treatment with CBD commonly causes transient side effects, it overall remains a safe treatment option [19]. 

‘Ablation’ and ‘VNS’ were both top keywords that have featured more prominently in recent years, indicating a rise in research related to laser ablation and VNS as treatment options for epilepsy. This could be due to their increasing acceptance in the medical field and recent technological advancements. Laser ablation involves using a laser to eliminate pathological areas of brain tissue. It is a less invasive treatment option compared with a standard craniotomy, but is not without risk, with 3.4% of children undergoing the procedure developing a 'severe' complication. Overall, more research has been deemed necessary to confirm its long-term efficacy [20]. VNS involves the implantation of a VNS therapy pulse generator and VNS therapy lead that attaches around and allows for stimulation of the vagus nerve [21,22]. In the USA, the Food and Drug Administration (FDA) first allowed the use of VNS for treating epilepsy in children in 1999. Since then, more studies have been carried out showing that VNS is possibly associated with a decreased seizure frequency in patients who are not suitable for surgical intervention [23,24]. Nonetheless, the post-implantation risk of infection is greater in children, and many studies involve predominantly adult participants, so further research is required to confirm these findings in a paediatric population [23]. 

Four out of the five most highly cited articles focus on alternative therapies for managing epilepsy, including the ‘ketogenic diet’, ‘cannabidiol-enriched cannabis’ use, and the ‘modified Atkins diet’. This increasing interest in complementary and alternative therapies has been demonstrated previously in the literature, with parents citing concerns about side effects of conventional medications and the perceived safety of dietary interventions as key reasons for exploring non-conventional medicine [25]. As well as this, although most individuals with epilepsy respond to treatment with anti-epileptic drugs, in 30% of patients, seizure control is not achieved [26]. The prevalence of drug-resistant epilepsy necessitates the investigation of alternative options for this group of children.

The only other bibliometric analysis done relating to epilepsy in children was an older review including articles from 1980 to 2018 [27]. This previous analysis included all articles related to paediatric epilepsy instead of focusing only on treatment options. Although some findings were replicated, such as the most productive country being the USA, others were not. During this earlier period, the most productive institution was found to be the Hospital for Sick Children, and the most productive journal was the Journal of Child Neurology. Neither of these featured in the top three for their respective categories in this analysis. This could be due to methodological differences and the slightly different scope of focus, but could also reflect genuine changes in this area of research over time.

One critique of this bibliometric analysis could be that too much emphasis is placed on quantitative aspects of research, such as the number of publications and citations, whilst not acknowledging the important qualitative aspects, such as originality and quality of each article. Another possible limitation is that the Web of Science database was the only database used. In future studies, data from multiple databases such as Scopus or PubMed could be combined to provide an even more comprehensive overview. As well as this, applying the search strategy to the title of documents only means that some relevant studies, including key terms only in the abstract, could be missed. Finally, excluding documents not published in English will likely cause an underestimation of the contribution to the field of non-English speaking authors and their respective countries.

## Conclusions

The increased morbidity and mortality associated with paediatric epilepsy mean that developing new treatment options for these children is of great importance. This bibliometric analysis examined 1135 documents published over the previous 20 years from 2005 to 2025 to identify important trends in paediatric epilepsy treatment. The analysis demonstrates that there has been a significant growth in research productivity, especially since 2018, with the USA leading the way both in terms of the number of publications and citations. International cooperation is also illustrated by Germany leading the way with the highest proportion of multiple-country publications.

The majority of the most highly cited articles were related to alternative therapies for managing epilepsy. Other key findings were the recent increase in research related to cannabidiol based treatments, vagal nerve stimulation, and laser ablation as therapeutic options. Through identifying these trends in the research and mapping author collaboration networks, it is hoped that this study can provide a foundational reference for researchers when planning future studies.
